# Health Tract, No. 114

**Published:** 1865

**Authors:** 


### HEALTH TRACT, No. 114.

### FIRE-PLACES.

Multitudes in large cities look back with fond regret to the gladsome days of childhood, when the blazing wood-fire on the hearth was but one of a multitude of other comforts and of other joys, departed now never to return — except the fire on the hearth! even the use of the old-fashioned open fire-place, with its fitful flickerings and its dancing shadows on the wall, to say nothing of its cheeriness, the pure air, and the delightfully soft and genial warmth which pervades the whole apartment. The writer has burned common hard or anthracite coal, flat on the hearth, in an old-fashioned, broad, deep fire-place, for three winters, consuming less than four tons of coal each, the fire burning from five A. M. to ten P. M. for nearly seven months. The extra heat warms a large chamber above sufficient to dress and undress for retiring in the very coldest weather. It also affords warmth enough to an adjoining room for the children practicing their music-lessons, and for ordinary social gatherings; this, too, when the furnace has not been fired-up once in two years, with an exemption from colds and loss of time at school (connected with colds) most gratifying to think of. When the halls are not warmed by furnace-heat, there are several very important advantages derived. The children especially get into a habit of briskness and activity in passing through them, largely promotive of a vigorous and active circulation of the blood and of good humor. This very activity of bodily motion which cold weather excites, instinctively seems to communicate its influences to the animal spirits and to the mental operations; all in striking contrast with that lazy, elephantine, drawling mode of locomotion and speech which settle down upon the inmates of a habitually summer-heated house.

In furnace-heated city houses the foul air of the cellar, and the hot and noisome fumes of the kitchen, fill the halls and chambers above; and in the latter they are breathed during the live-long night, to the inevitable enfeeblement of the constitution and the ultimate destruction of the health. The most ignorant know that sudden changes of air are hurtful, even perilous, in proportion to their intensity and frequency; inducing those pneumonias, inflammation of the lungs, or lung fevers, which have been so frequently and speedily fatal in multitudes of cases; and even when recovered from, are attended with a painful and tedious convalescence, extending sometimes to years, and not seldom crippling the health for the remainder of life. Furnaces are commonly arranged to keep up a heat of near seventy degrees Fahrenheit.

On the first day each of last December, January, February, and March, our thermometer, at six in the morning, stood at 36, 32, 28, 28, respectively, making an average difference of 40 degrees between the out and in-door temperature. But one fourth that difference, from 20 down to 10, above zero, is piercingly felt in winter, hence a sudden change so great as 40 degrees must greatly imperil the health and even life of the old, of the feeble, and of young children; but these dangers must be greatly lessened in passing from a sitting-room of 70, through a hall of 50, into the out-door air of 30, as would be the case where open fire-places are used instead of furnaces. A piece of soft coal or light-wood, six inches square, laid on a bed of coals, in a low-down grate, will give the beautiful and cheery flickering of the old-fashioned fire-place; and then there are all the advantages of the open fire as to constant ventilation and genial warmth.

